# Rare Variants Association Analysis in Large-Scale Sequencing Studies at the Single Locus Level

**DOI:** 10.1371/journal.pcbi.1004993

**Published:** 2016-06-29

**Authors:** Xinge Jessie Jeng, Zhongyin John Daye, Wenbin Lu, Jung-Ying Tzeng

**Affiliations:** 1 Department of Statistics, North Carolina State University, Raleigh, North Carolina, United States of America; 2 Epidemiology and Biostatistics, University of Arizona, Tucson, Arizona, United States of America; 3 Bioinformatics Research Center, North Carolina State University, Raleigh, North Carolina, United States of America; 4 Department of Statistics, National Cheng-Kung University, Tainan, Taiwan; Indiana University, UNITED STATES

## Abstract

Genetic association analyses of rare variants in next-generation sequencing (NGS) studies are fundamentally challenging due to the presence of a very large number of candidate variants at extremely low minor allele frequencies. Recent developments often focus on pooling multiple variants to provide association analysis at the gene instead of the locus level. Nonetheless, pinpointing individual variants is a critical goal for genomic researches as such information can facilitate the precise delineation of molecular mechanisms and functions of genetic factors on diseases. Due to the extreme rarity of mutations and high-dimensionality, significances of causal variants cannot easily stand out from those of noncausal ones. Consequently, standard false-positive control procedures, such as the Bonferroni and false discovery rate (FDR), are often impractical to apply, as a majority of the causal variants can only be identified along with a few but unknown number of noncausal variants. To provide informative analysis of individual variants in large-scale sequencing studies, we propose the Adaptive False-Negative Control (AFNC) procedure that can include a large proportion of causal variants with high confidence by introducing a novel statistical inquiry to determine those variants that can be confidently dispatched as noncausal. The AFNC provides a general framework that can accommodate for a variety of models and significance tests. The procedure is computationally efficient and can adapt to the underlying proportion of causal variants and quality of significance rankings. Extensive simulation studies across a plethora of scenarios demonstrate that the AFNC is advantageous for identifying individual rare variants, whereas the Bonferroni and FDR are exceedingly over-conservative for rare variants association studies. In the analyses of the CoLaus dataset, AFNC has identified individual variants most responsible for gene-level significances. Moreover, single-variant results using the AFNC have been successfully applied to infer related genes with annotation information.

## Author Summary

Next-generation sequencing technologies have allowed genetic association studies of complex traits at the single base-pair resolution, where most genetic variants have extremely low mutation frequencies. These rare variants have been the focus of modern statistical-computational genomics due to their potential to explain missing disease heritability. The identification of individual rare variants associated with diseases can provide new biological insights and enable the precise delineation of disease mechanisms. However, due to the extreme rarity of mutations and large numbers of variants, significances of causative variants tend to be mixed inseparably with a few noncausative ones, and standard multiple testing procedures controlling for false positives fail to provide a meaningful way to include a large proportion of the causative variants. To address the challenge of detecting weak biological signals, we propose a novel statistical procedure, based on false-negative control, to provide a practical approach for variant inclusion in large-scale sequencing studies. By determining those variants that can be confidently dispatched as noncausative, the proposed procedure offers an objective selection of a modest number of potentially causative variants at the single-locus level. Results can be further prioritized or used to infer disease-associated genes with annotation information.

This is a *PLOS Computational Biology* Methods paper.

## Introduction

Recent advances in next-generation sequencing (NGS) technologies have extended the focus of genetic studies of complex traits from that of common to rare variants. Having low minor allele frequencies (MAFs), usually defined to be less than 1% to 5%, rare variants are often evolved from recent mutations that have not yet been subjected to the pruning mechanism of natural selection and can potentially retain a larger proportion of inheritable variability than common variants. [1–5] Recent studies have already implicated the relevance of rare variants on several complex traits. [6–13]

Despite its potential to uncover genetic factors contributing to missing disease heritability, the analysis of rare variants association studies bears fundamental challenges. As only a small proportion of samples may carry variant alleles at each locus, associations of individual rare variants are often underpowered. [1, 14, 15] Moreover, the number of candidate variants can be extremely large in high-throughput sequencing studies, in which available multiple testing strategies may impose excessively severe corrections, preventing the selection of potentially causal variants. [16]

Recent proposals for rare variants association analysis often resort to collapsing or pooling multiple variants in a gene or pathway. Examples include the combined multivariate collapsing (CMC) [17], cohort allelic sum (CAST) [18], C-alpha [19], sum of squared scores [20–23], sequence kernel association (SKAT) [24], quality-weighted multivariate score association (qMSAT) [25], and similarity-based regression (simReg) [26] tests. The strategy increases power by aggregating effects of low-frequency variants and decreasing data dimension in multiple testing. It has been successfully applied in several applications that identified functional regions that may contain potentially relevant rare variants. [17–20, 23–26]

Nonetheless, variants-pooling tests that aggregate over a gene or pathway do not provide information at the individual locus and are ill-equipped to tap the full potential of NGS data in identifying causative mutations at the single-nucleotide resolution. Pinpointing potentially causal variants is a critical goal of genomic studies because such information would faciliate precise delineations of molecular mechanisms and functions of genetic factors on diseases. [27] Moreover, studies have shown that pooling over multiple variants may result in reduced power, as the inclusion of many noncausal variants may dilute the effects of relevant variants on a trait. [28–30] Thus, pooling over multiple variants can sometimes be inadequate for the identification of functional genomic regions.

On the other hand, analysis of individual rare variants can provide practical advantages. Information of single-variant association can be used to pinpoint a small number of potentially causal variants for follow-up studies to facilitate the precise characterization of functions via molecular modeling and genetic experimentation, which are often too expensive and time consuming to conduct for all variants in a gene. [27] Further, single-variant results can be utilized *a posteriori* to objectively infer disease-related genes or pathways by comparing with annotation and functional databases. [31–34] This is useful as gene-level results can oftentimes be uninformative when the significance of a few causal variants are diluted by a large number of noncausal ones in the same gene. In the Results section, we will illustrate both strategies for applying single-variant results using the CoLaus data set.

Genome-wide association (GWA) studies, as the pre-eminent means for genetic discovery over the last decade, have largely relied on statistical genomic tools that can identify common variants at the individual single-nucleotide polymorphism (SNP) level. [35] Standard procedures for GWA studies evaluate each variant individually. [36, 37] Potentially causal variants are identified by multiple-testing control on significances at each locus. The simplest strategy for multiple testing utilizes the Bonferroni correction that controls family-wise error rate, or the probability of having one or more false positives. [38] However, the Bonferroni correction can often be too conservative for GWA studies under the presence of thousands of SNPs. [39] To address this issue, the false discovery rate (FDR) is often utilized that provides a more liberal criterion by controlling the expected proportion instead of the presence of false positives. [40–42]

**Fig 1:**
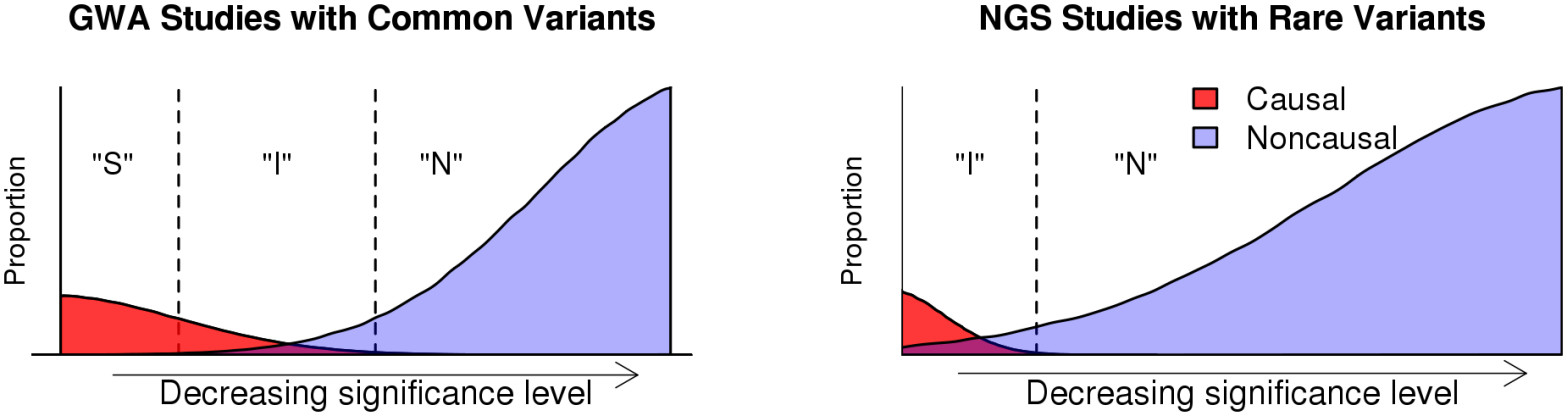
Illustrations of regions of statistical inference for GWA and NGS studies. The Signals (“S”), Indistinguishable (“I”), and Noise (“N”) regions are shown. False-positive control allows the selection of variants in the Signals region, whereas false-negative control selects from both the Signals and Indistinguishable regions. In NGS studies with rare variants, the Signals region often degenerates due to extremely low MAF and high dimensionality.

Despite being extremely successful for common variants in GWA studies [43–46], procedures based on false-positive control are often underpowered in NGS studies involving rare variants (as illustrated in Fig 1). New approaches are needed to provide a meaningful way for powerful variants selection in large-scale sequencing studies. Fig 1 compares the statistical landscape of rare variants analysis in NGS studies with that of common variants in GWA studies. In GWA studies, we observe three regions of statistical inference: the Signals (“S”) region where strongly associated variants can be readily identified by controlling false positives, the Noise (“N”) region where noncausal variants can be identified by controlling false negatives, and the indistinguishable (“I”) region where causal and noncausal variants are inextricably mixed. [47, 48] We have recently developed theoretical characterizations for the three regions in high-dimensional data analysis. [49] In NGS studies with rare variants, the Signals region tends to be very narrow and can often degenerate due to extremely low MAF and high dimensionality. Consequently, few causal variants can be identified by evaluating false positives, and results can be very unstable due to random perturbations of noncausal variants.

To address the challenge of rare variants association analysis at the single-locus level, we propose the Adaptive False-Negative Control (AFNC) procedure in order to allow a large proportion of causal variants to be retained with high probability. Specifically, the AFNC applies a novel metric called the signal missing rate (Eq 2), defined as the probability of having a nontrivial proportion of false negatives among all causal variants (i.e., FN/*s* in Table 1), to achieve informative variant selection by controlling the signal missing rate to be small (see Methods section). That is, AFNC seeks to determine those variants that can be confidently dispatched as noncausal and identifies variants from both the Signals and Indistinguishable regions. The results can provide informative inference in NGS studies where the Signals region is very small or degenerate (Fig 1).

**Table 1:** Classifications of variants under multiple testing control.

	Selected	Not selected	Total
**Causal**	TP	FN	*s*
**Noncausal**	FP	TN	*d* − *s*
	*R*	*d* − *R*	*d*

TP, FN, FP, and TN are numbers of true positives, false negatives, false positives, and true negatives, respectively. *R* is the number of variants selected.

We note that this is quite different from classical methods that control false positives. For example, the Bonferroni controls for the presence of any false positives (i.e., FP ≥ 1), whereas the FDR controls for the expectation of the proportion FP/*R* when *R* > 0 (see Table 1). Neither of these involve the number of causal variants *s*; thus, they cannot be used for controlling the proportion of causal variants selected. On the other hand, the AFNC, based on the proportion FN/*s* or 1 − TP/*s*, allows powerful variants selection by controlling the type II error or 1 − statistical power. Although there may exist a corresponding control level for the FDR (albeit very large) that can include the variants selected by the AFNC at a given false-negative control level (see Results section), this corresponding FDR control level is not known *a priori* and is expected to vary haphazardly across different studies. An arbitrarily assigned FDR control level would be inefficient for controlling false negatives in NGS studies, that can over- or under-select uncontrollably depending on the size of the Noise region. A corresponding control level usually does not exist for the stringent Bonferroni selection in large-scale sequencing studies (see Results section).

The AFNC provides a general framework that can accommodate for a wide spectrum of models and test statistics, that may include biological prior knowledge and global genotype information (see Methods section). Moreover, it readily adapts to the quality of statistical tests employed. With improved quality of statistical tests, the Indistinguishable region (see Fig 1) narrows, and the AFNC can, in turn, select a smaller set of potentially causal variants. Extensive studies (see Results section) demonstrate that the AFNC can identify a modest number of potentially causal variants while avoiding a deluge of noncausal ones for follow-up analyses that focus on targeted variants. Our proposal employs recent developments in ultra high-dimensional statistical inference to derive a data-driven procedure that can readily adapt to the underlying sparsity and effect sizes of the data. [50–53] It readily controls type I error rates (see Results section). In addition, it is computationally very efficient and can be applicable for whole-genome sequencing (WGS) and whole-exome sequencing (WES) studies.

## Results

The AFNC provides a general framework for including a high proportion of causal variants. It can accommodate for a spectrum of models and significance tests. The procedure (detailed in the Methods section) consists of three major steps: (i) based on a given model and significance test, obtain the test statistics and their *p*-values for each of the *d* variants and order them, (ii) estimate the signal proportion among the *d* variants (denoted by $\hat{\pi }$) using Eq 4, and (iii) compute the AFNC cut-off position ${\hat{T}}_{fn}$ by controlling the signal missing rate at level *β* using Eq 3 and report the top ${\hat{T}}_{fn}$ variants as potentially causal. The AFNC is designed to allow researchers to select a modest number of potential variants while encompassing the causal ones with high confidence. Below we use simulation studies and data applications to illustrate the utility of AFNC.

### Simulation studies

#### Simulation designs

We obtained 10,000 haplotypes for a 25Mb region simulated by COSI 1.2 (http://www.broadinstitute.org/~sfs/cosi) according to a coalescent model that emulates the linkage-disequilibrium (LD) pattern and history of the European population using default parameters. [54] For each subject *i*, *i* = 1, ⋯, *n*, we randomly drew two haplotypes with replacement from the 10,000 haplotypes to form its genotypes *G*_*ij*_ across variants *j* = 1, ⋯, *d*, where we assumed an additive genetic model such that *G*_*ij*_ ∈ {0, 1, 2} is the number of minor alleles at locus *j*. For an experiment with sample size *n*, we focused on evaluating rare variants with $0<\text{MAF}<1/\sqrt{2n}$, where the threshold $1/\sqrt{2n}$ was derived from statistical theory and has been employed in providing principled demarcations of rare and common variants in recent literature. [52, 53, 55] It incorporates sample-size information of individual experiments to determine if a variant is rare. For example, a variant with 1% MAF will be considered rare in an experiment when *n* = 2000 and common when *n* = 10,000. There were at least 250,000 numbers of rare variants with $0<\text{MAF}<1/\sqrt{2n}$ for randomly generated data at sample sizes *n* = 1000, 2500, 5000, 7500, and 10,000. These variants were truncated to obtain subsets of the data with different numbers of total variants *d* in various simulation scenarios. We randomly generated phenotypes in each experiment from the Normal distribution $Y_{i}∼N\left(\sum_{j=1}^{s}G_{ij}A_{j},\sigma^{2}\right)$, where *s* is the number of causal variants, *A*_*j*_ is the effect size of the *j*th locus, and *σ* is the noise level fixed at 1. We selected the first *s* variants as causal so that the causal variants in different simulation scenarios are nested. As in previous studies, we set the effect sizes *A*_*j*_ = *C*⋅|log_10_(*MAF*_*j*_)| for variants *j* = 1, …, *s* and 0 otherwise. [24] Thus, a continuum of effect sizes can be shown by varying the effect-size multiplier *C*.

The AFNC was compared with the Bonferroni and FDR controls, which are the most commonly used procedures for adjusting multiplicity in genomic studies. Bonferroni controls the family-wise type I error [38], whereas FDR controls the expected proportion of false positives among all discoveries [41]. Both essentially focus on the control of false positives with FDR being less stringent than the Bonferroni. The Bonferroni and FDR threshold levels were both set at 0.05. The AFNC threshold levels were set at a false-positive rate of *α* = 0.05 and a false-negative rate of *β* = 0.1. When estimating *π* in Step (ii) of AFNC (Eq 4), the *c*_*d*_ values, obtained from Eq 5, are 0.0488, 0.0305, 0.0150, and 0.0095 for *d* = 10,000, 25,000, 100,000, and 250,000, respectively, based on *M* = 10,000 randomly generated samples under the global null hypothesis of no causal variants.

For succinct presentation, we compared the AFNC with the Bonferroni and FDR using the Wald test. In the following, we illustrate that the AFNC can perform well, even though significance rankings based on the Wald test may not be optimal. Performances were comprehensively evaluated via sensitivity, specificity, and *g*-measure [56], and success rates of inclusion of a high proportion of causal rare variants. Sensitivity is defined as the proportion of causal variants that were correctly identified and provides the empirical power for *s* > 0 causal variants. Specificity is the proportion of noncausal variants that were correctly rejected. Under the global null hypothesis when all variants are noncausal (i.e., *C* = 0), the empirical type I error rate or false-positive rate is defined as 1—specificity. The *g*-measure, defined as $\sqrt{\text{sensitivity}\cdot \text{specificity}}$, is a composite performance measure of overall variant selection. [56, 57] A *g*-measure close to 1 indicates accurate variant selection, and a *g*-measure close to 0 implies that few causal variants or too many noncausal ones are selected, or both. Each experimental scenario was randomly simulated 100 times. Median results are shown for sensitivity, specificity, and *g*-measure, whereas success rates of inclusion of at least a given proportion of causal variants were computed based on the 100 repetitions.

#### Comparison across different effect sizes and numbers of variants

We evaluated performances across varying numbers of total variants *d* and effect-size multipliers *C*. We considered *s* = 50 variants, which are causal when *C* ≠ 0. Experiments were conducted with *n* = 2000 number of samples.

**Fig 2:**
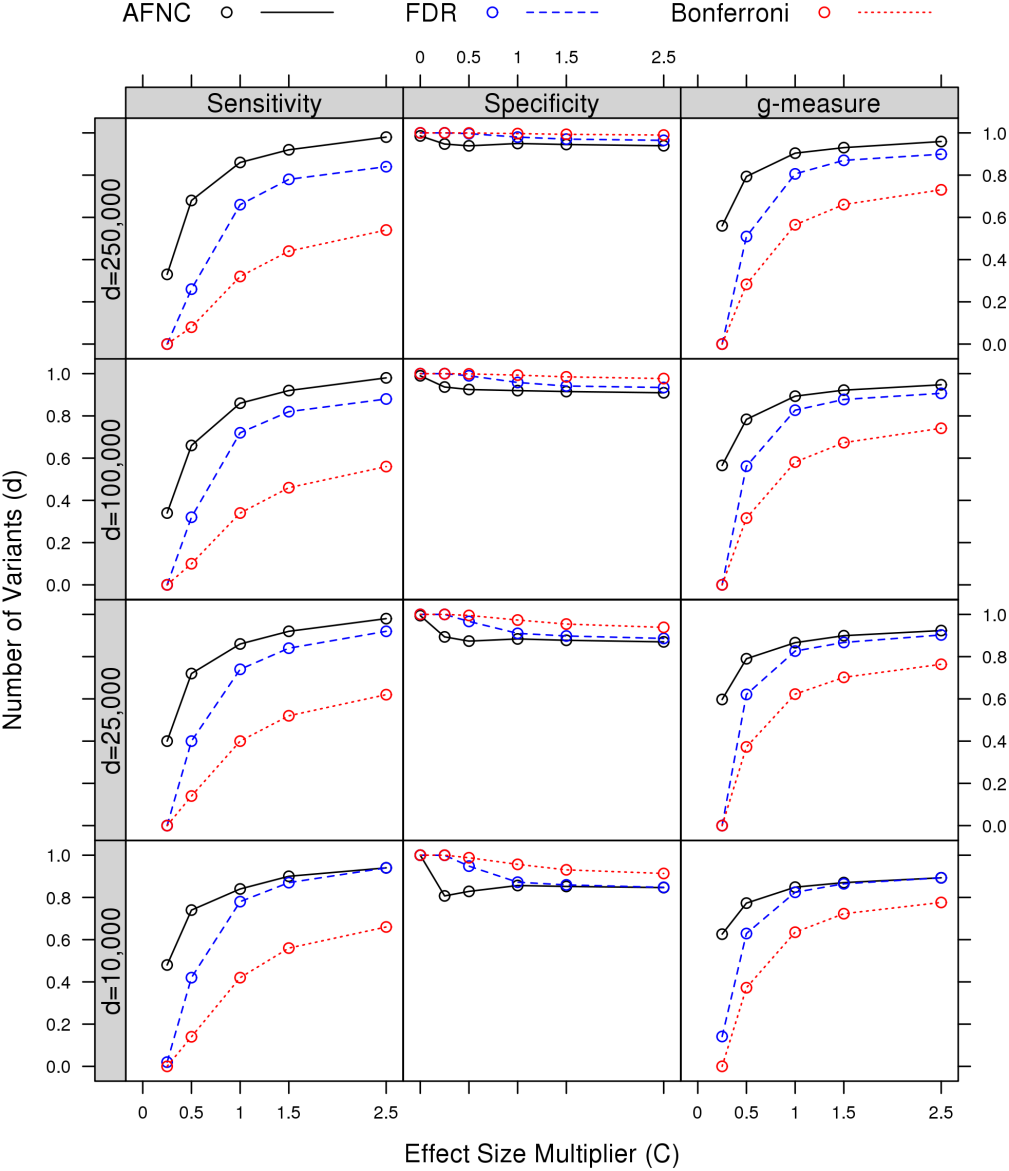
Comparisons across varying effect sizes and numbers of variants at *s* = 50. Performance of AFNC, FDR, and Bonferroni is evaluated in terms of sensitivity, specificity, and *g*-measure. Results are shown for *s* = 50 number of causal variants when *C* ≠ 0 and *n* = 2000 number of samples.

Fig 2 presents results of sensitivity, specificity, and *g*-measure. The AFNC consistently dominates the FDR and Bonferroni across numbers of variants *d* and effect-size multipliers *C* in terms of sensitivity or empirical power for *C* ≠ 0. Success rates of including at least a given proportion of the *s* causal variants are presented in S1 Fig. AFNC successfully selects at least 75% of causal variants when *C* is relativley large, whereas FDR and Bonferroni usually cannot select a large proportion of causal variants, especially for *d* large. In fact, the Bonferroni fails to select more than 75% of causal variants in all scenarios. This suggests the advantage of considering false-negative control procedures over false-positive ones for including causal rare variants.

AFNC underperforms the Bonferroni and FDR in terms of specificity in Fig 2. Nonetheless, AFNC consistently dominates the Bonferroni and FDR in terms of overall performances with the *g*-measure, especially at *d* large. This suggests that the AFNC can improve overall variant-selection performance in large-scale sequencing studies. Specifically, the AFNC, at the cost of mildly increased but controlled false positives, provides dramatic reduction in the number of candidate variants while retaining a high proportion of causal ones for follow-up analysis. However, variant screening with the AFNC comes with a cost. Although AFNC selects a small proportion of variants, the actual number of selected variants can be large in high dimensions, which can result in severely lower precision (i.e., the proportion of true positives among those selected, *TP*/*R*) compared with the Bonferroni and FDR.

Table 2 presents empirical type I error rates at the global null hypothesis *C* = 0 when no variants are causal. The AFNC is shown to control type I error rates well at below *α* = 0.05. This is due to the adaptivity of the AFNC procedure that allows it to accommodate for varying proportions of causal variants (see Methods section). On the other hand, Bonferroni and FDR have type I error rates at 0, suggesting them to be much too conservative for rare-variant association studies.

**Table 2:** Empirical type I error rates across varying numbers of variants.

Number of variants	Bonferroni	FDR	AFNC
*d = 10,000*	0 (0)	0 (4.03 × 10^−4^)	0 (0.092)
*d = 25,000*	0 (0)	0 (0.002)	0.006 (0.053)
*d = 100,000*	0 (0)	0 (0)	0.010 (0.030)
*d = 250,000*	0 (4.00 × 10^−7^)	0 (1.96 × 10^−5^)	0.014 (0.032)

Standard errors are included in parentheses. Results are shown at the sample size *n* = 2000.

We repeated the same evaluation with *s* = 25 variants, which are causal when *C* ≠ 0. Results are presented in S2 Fig (for sensitivity, specificity, and *g*-measure) and S3 Fig (for success rates of inclusion). The relative performance among AFNC, FDR, and Bonferroni is similar to what is observed for *s* = 50.

#### Comparison across different sample sizes and numbers of causal variants

We compared performances across different sample sizes *n* and numbers of causal variants *s*. An effect-size multiplier *C* = 0.5 is considered at *d* = 100,000 total number of variants.

**Fig 3:**
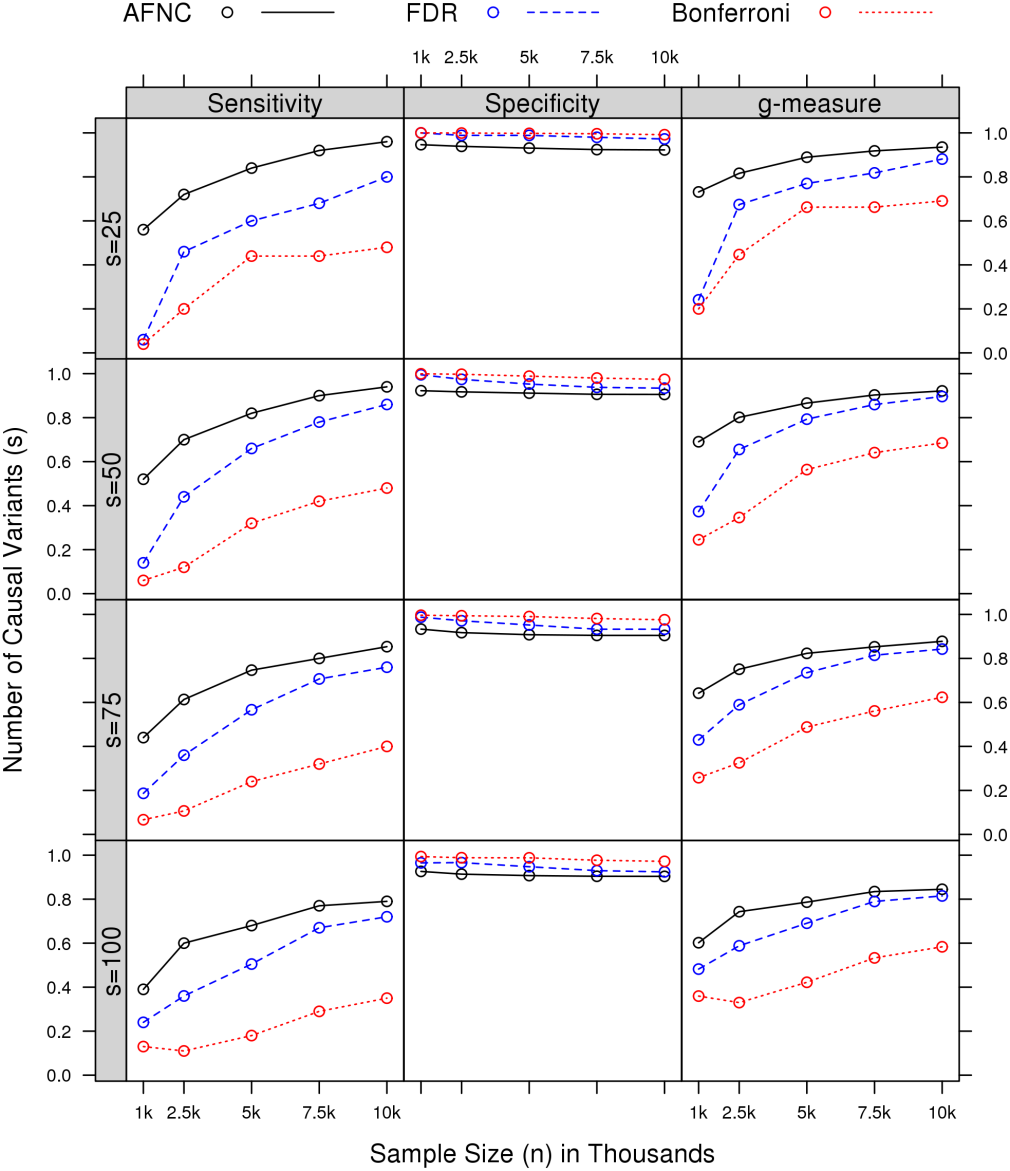
Comparisons across varying sample sizes and numbers of causal variants at *C* = 0.5. Performance of AFNC, FDR, and Bonferroni is evaluated in terms of sensitivity, specificity, and *g*-measure. Results are shown for the effect-size multiplier *C* = 0.5 and *d* = 100,000 number of variants.

Fig 3 shows that the AFNC consistently outperforms the FDR and Bonferroni across numbers of causal variants *s* and sample sizes *n* in terms of sensitivity or empirical power. Success rates of inclusion are shown in S4 Fig, where the AFNC can select at least 75% of causal variants for sample size *n* large. The FDR and Bonferroni usually select a small proportion of causal variants with the Bonferroni consistently selecting less than 50% of causal variants in nearly all scenarios. Due to low MAFs, selection of causal variants is more difficult for rare variants at small sample sizes. For example, at *n* ≤ 2500, the procedures usually cannot identify more than 90% of all causal variants. Fig 3 shows that the AFNC dominates the FDR and Bonferroni for overall variant selection in terms of *g*-measure with underperformance in terms of specificity. Moreover, S1 Table presents empirical type I error rates at varying sample sizes *n*, where the AFNC is shown to control type I error rates at 0.05 while the FDR and Bonferroni are overwhelmingly over-conservative with type I error rates at 0. S5 and S6 Figs further present results at *C* = 0.25, where the AFNC is shown to be even more advantageous at smaller effect sizes.

### Analysis of CoLaus cardiovascular diseases dataset

We considered the Cohorte Latusannoise (CoLaus) sequence study [58–61], where almost 6000 unrelated Caucasian residents of Lausanne, Switzerland were assessed for risk factors of cardiovascular diseases (CVD). Targeted sequencing genotypes on 202 drug-targeted genes (human genome build 36) were obtained for *n* = 1769 of these subjects. Cholesterol levels were collected for each subject to evaluate risk of CVD, along with 12 clinical factors—age, gender, and 10 ethnicity covariates using the first 10 principal components [62]. We considered *d* = 9665 autosomal rare variants from the sequencing study with $0<MAF<1/\sqrt{2n}=0.0072$.

For each variant, *t*-statistic was obtained by linear association with log cholesterol levels as the response while adjusting for the 12 clinical covariates. The AFNC, FDR, and Bonferroni were, then, applied on significances of *t*-statistics to identify potentially causal variants. At threshold levels of 0.05, Bonferroni and FDR only identified 4 variants. At *α* = 0.05 and *β* = 0.1, AFNC identified 56 candidate rare variants. The AFNC algorithm obtained *c*_*d*_ = 0.0494 based on *M* = 10,000 randomly generated samples under the global null of no causal variants and $\hat{\pi }=0.001784$ (Eqs 4 and 5). As CVD tends to be influenced by multiple factors [63, 64] and the study focused on genes having clinical relevance, one expects a larger number of causal variants than those identified by the FDR and Bonferroni. Our estimated number of signals, $\hat{s}=\hat{\pi }\times 9665=17.244$, suggests that at least 18 variants need to be selected, and potentially much more due to signals dispersed in the Indistinguishable region, to encompass a high proportion of causal variants. That is, false-positive control procedures can be much too conservative in NGS studies, where the Signals region tends to be degenerate (see Fig 1). In the following, we illustrate potential applications of the AFNC for pinpointing individual variants in candidate genes and inferring disease-related genes with annotation information.

#### Pinpointing individual variants in candidate genes for follow-up analysis

To obtain a set of candidate genes, we conducted gene-based analysis using the SKAT with the linear kernel and variant weights 1/MAF. [24] The SKAT performs gene-level analyses via variance component test. The SKAT with the linear kernel is equivalent to the SimReg [26] and the sum of squared scores [20–23] tests. Gene-based analysis did not identify any significant gene when controlling the FDR at the 0.05 threshold level. For illustrative purposes, we focused on the top 5 genes (*APH1A*, *TRPM8*, *SLC10A2*, *SP110*, *SIRT6*) with gene-set *p*-values <0.01. These genes have been related to CVD in the literature. [65–74]

Table 3 presents variants selected in the top 5 candidate genes by the AFNC, along with their *p*-values and annotation information. The Bonferroni and FDR only selected 2 variants, chr1_148504677 from *APH1A* and chr2_234559154 from *TRPM8*. They did not identify any variant from *SP110* and *SIRT6*. Both are relevant genes, where *SP110* has been associated with venous obstruction [67] and *SIRT6* has been known for its therapeutic potential towards the prevention of CVD [72–74]. Moreover, *TRPM8*, from which the FDR and Bonferroni only identified a single variant, regulates functions of the pulmonary artery via complex systems. [68–70] No individual variants were selected from *SLC10A2*, whose most significant variant has a *p*-value of 6.32 × 10^−3^.

**Table 3:** Annotation of AFNC-selected variants of candidate genes in the analysis of CoLaus data.

Gene (gene-set *p*-value)	Variant ID	Variant *p*-value	Variant type
*APH1A* (1.90 × 10^−3^)	*chr1_148504677	5.15 × 10^−6^	downstream
*TRPM8* (3.54 × 10^−3^)	*chr2_234559154	5.15 × 10^−6^	non-synonymous coding
	chr2_234543736	6.21 × 10^−5^	non-synonymous coding
	chr2_234556441	6.44 × 10^−4^	synonymous coding
	chr2_234591833	6.63 × 10^−4^	downstream
*SP110* (4.14 × 10^−3^)	chr2_230785852	6.12 × 10^−4^	non-synonymous coding
	chr2_230745800	1.17 × 10^−3^	splice site
*SIRT6* (6.68 × 10^−3^)	chr19_4125175	2.06 × 10^−4^	3’ UTR

Gene-set *p*-values are computed using the SKAT. Genes are sorted in increasing gene-set *p*-values, and variants are sorted by their individual *p*-values among each gene. Variants marked with (*) are also selected by the Bonferroni and FDR at the 0.05 level.

The AFNC, based on global hypothesis tests, provides an objective selection of a modest number of potentially causal variants at the single-locus level. Investigators may further prioritize variants using annotation information. For example, in Table 3, one may first target variants at non-synonymous coding and splice sites that can disrupt protein structures before analyzing 3’/5’ UTR and downstream/upstream variants that may regulate gene expression. [75] Synonymous coding and intron variants may also impact gene expression, protein folding, and fitness. [76–78] Nonetheless, they are usually considered as low-priority and may represent irrelevant variants that were mixed indistinguishably with the causal ones due to extremely low MAF and high dimensionality.

#### Inferring disease-related genes with single-variant results

Gene-based analysis using variants pooling can sometimes result in limited power due to the inclusion of many noncausal variants. For example, gene-set analysis using the SKAT did not identify any candidate genes in this study when controlling the FDR at the 0.05 level on gene-set *p*-values for risk of CVD, a multifaceted disease. To provide an alternative approach, we consider the utilization of single-variant results to infer candidate genes. Specifically, among the 56 AFNC variants, we further focus on non-synonymous and splice-site variants that are often considered as prime candidates for causal variants due to their capacity to influence protein coding and structure. [75] Table 4 presents non-synonymous and splice-site variants selected. The Bonferroni and FDR only selected a single variant, chr2_234559154 from *TRPM8*, whereas the AFNC selected 16 variants from 14 genes. The number of non-synonymous and splice-site variants selected by AFNC is at the same magnitude as our estimated number of causal variants $\hat{s}=17.244$. *SP110* and *TRPM8*, that contain 2 AFNC-selected non-synonymous and splice-site variants, have been related to venous obstruction [67] and pulmonary functions [68–70], respectively. Moreover, genes with a AFNC-selected non-synonymous or splice-site variant have been associated with CVD (*BRD2* [79], *CNR2* [80–82], *KCNN4* [83–86], *MME* [87, 88], *NLRP1* [89], *SDHB* [90], *TACR3* [91, 92], *TNNI3K* [93–95]) or related conditions, such as diabetes (*CLEC16A* [96]), obesity (*OPRM1* [97, 98]), chronic obstructive pulmonary disease (*PDE4A* [99–102]), and diabetic peripheral neuropathy (*SCN9A* [103, 104]). The full annotation of FDR- and AFNC-selected variants are shown in S2 Table.

**Table 4:** Annotation of AFNC-selected non-synonymous and splice-site variants in the analysis of CoLaus data.

Gene (gene-set *p*-value)	Variant ID	Variant *p*-value	Variant type
*BRD2* (0.281)	chr6_33053682	2.08 × 10^−3^	non-synonymous coding
*CLEC16A* (0.0902)	chr16_11125133	2.06 × 10^−4^	non-synonymous coding
*CNR2* (0.139)	chr1_24073736	2.27 × 10^−3^	non-synonymous coding
*KCNN4* (0.456)	chr19_48965473	6.44 × 10^−4^	non-synonymous coding
*MME* (0.387)	chr3_156315473	1.03 × 10^−3^	splice site
*NLRP1* (0.303)	chr17_5425965	2.73 × 10^−3^	non-synonymous coding
*OPRM1* (0.627)	chr6_154454129	5.06 × 10^−4^	non-synonymous coding
*PDE4A* (0.313)	chr19_10439268	2.06 × 10^−4^	non-synonymous coding
*SCN9A* (0.291)	chr2_166845210	6.21 × 10^−5^	non-synonymous coding
*SDHB* (0.674)	chr1_17232220	9.24 × 10^−4^	non-synonymous coding
*SP110* (4.14 × 10^−3^)	chr2_230785852	6.12 × 10^−4^	non-synonymous coding
	chr2_230745800	1.17 × 10^−3^	splice site
*TACR3* (0.0149)	chr4_104859945	2.06 × 10^−4^	non-synonymous coding
*TNNI3K* (0.537)	chr1_74701758	1.25 × 10^−3^	non-synonymous coding
*TRPM8* (3.54 × 10^−3^)	*chr2_234559154	5.15 × 10^−6^	non-synonymous coding
	chr2_234543736	6.21 × 10^−5^	non-synonymous coding

Gene-set *p*-values are computed using the SKAT. Genes are sorted in alphabetic order, and variants are sorted by their individual *p*-values among each gene. Variants marked with (*) are also selected by the Bonferroni and FDR at the 0.05 level.

#### Comparison with Bonferroni and FDR at varying control levels

Table 5 presents numbers of variants selected by the Bonferroni and FDR at different control levels. The Bonferroni, based on the stringent family-wise type I error rate, cannot select more than 10 variants even at the maximum control level of 1. That is, when more than 10 variants are selected, a false positive will almost surely be included with probability 1. In this particular analysis, FDR at the 0.55 control level can select the 56 variants obtained by the AFNC at *α* = 0.05 and *β* = 0.1. However, we note that the FDR control level corresponding to the AFNC is not invariant and can vary dramatically across different studies. Intuitively, a larger (or smaller) FDR control level would be needed when the Indistinguishable region is larger (or smaller) (see Fig 1), and this cannot be determined *a priori*.

**Table 5:** Number of variants selected in the analysis of CoLaus data at different control levels.

Control level	0.01	0.05	0.5	0.9	0.99	1
Bonferroni	0	4	4	10	10	10
FDR	0	4	45	493	7442	9665

At each level, Bonferroni controls the family-wise type I error, whereas the FDR controls the expected proportion of false positives among all discoveries.

## Discussion

We have proposed a novel bioinformatic approach that allows the identification of individual rare variants in large-scale sequencing association studies. Extensive studies based on simulated data generated with COSI at realistic population parameters have been used to compare our method with the Bonferroni and FDR across various scenarios. [54] Results have suggested that the AFNC can provide informative variant selection by including a large proportion of causal variants while avoiding a deluge of noncausal ones. On the other hand, the Bonferroni and FDR are shown to be excessively over-conservative under extremely low MAFs and high dimensionality. Analyses of the CoLaus dataset for cardiovascular diseases using the AFNC have pinpointed individual variants most responsible for explaining significances of genes identified in gene-level aggregation tests. Moreover, single-variant results have been successfully applied to objectively infer potentially relevant genes when cross-referenced with annotation information. The R package ‘AFNC’ for performing the AFNC is publicly and freely available at https://github.com/zjdaye/AFNC or http://sites.google.com/site/zhongyindaye/software.

The AFNC provides a unified framework to accommodate for a wide spectrum of models, test statistics, and data scenarios. To achieve a succinct presentation, we focused on quantitative traits using *p*-values obtained from linear association tests in this paper. The AFNC can be easily adopted for case-control studies [23–25, 105], family-structured data [106, 107], and many other scenarios. Moreover, empirical *p*-values, as from permutation or bootstrap, can be employed for improved significance ranking. [108] Clearly, performance results of the AFNC using *p*-values based on associations with quantitative traits, shown in this paper, can be extended to those obtained under a spectrum of models and data scenarios. Moreover, the analysis of large-scale genomic data is a dynamic and fast-evolving field. The AFNC, that readily adapts to the quality of statistical tests employed, will be able to provide increasingly efficient inclusion of causal variants as ever more accurate and computationally efficient means for assessing significances are developed.

A few very recent works have sought to identify individual rare variants by incorporating prior-knowledge information in statistical inference. [109, 110] These methods typically upweight individual variants predicted to be most likely to be causal based on prior GWA studies, functional annotation, sequence conservation, and other computational means. The AFNC can be readily utilized with models and test statistics that incorporate biological prior knowledge. In the Results section, we illustrated an alternative way to incorporate this bioinformatic knowledge. Specifically, we started with an agnostic interrogation of each variant and obtained a set of statistically promising variants using AFNC. We then compared the selected variants with prior-knowledge information to allow investigators to form educated hypothesis in designing follow-up studies. Statistically promising variants, that are selected objectively by AFNC, can also be explored in follow-up studies without comparing with annotation information, such as when prior knowledge is not available for novel variants or believed to be inaccurate.

Due to extremely low MAFs, rare variants do not usually exhibit strong linkage disequilibrium. [1, 111] Thus, we designed the AFNC for rare variants association studies, in which dependence among test statistics is assumed to be weak. The AFNC procedure is also applicable in the situation when causal variants are dependent, but noncausal variants are independent. [112] In other applications where noncausal genetic factors are expected to be strongly dependent, the AFNC procedure can be adapted to account for arbitrary dependence using several recent techniques for multiple testing. [113, 114]

One potential limitation of AFNC is that it may underperform when the signal intensity of the causal variants is too low. The signal intensity of a causal variant depends on the effect sizes and sample size. As shown in Figs 2 and 3, the sensitivity of AFNC deteriorates as effect size or sample size becomes smaller. Indeed, low effect sizes and small sample size are fatal limitations to all methods. In single-variant analysis of rare variants, such challenges may arise from identifying the extremely rare causal variants (e.g., singletons in the data). Although effect size is believed to be high for rare causal variants, the overall signal intensity may still be low given the extremely low sample size. Under this scenario, gene-based tests coupled with functional annotation would have better potential to identify these causal variants. Therefore, gene-based tests, functional annotation and AFNC should be used in an integrated fashion in the detection of rare causal variants: as we have illustrated in our analysis of the CoLaus data, AFNC coupled with gene-based tests can help to pinpoint potential causal variants that lead to gene-level significance; AFNC coupled with functional annotation can help to identify causal genes that are insignificant at gene level due to a few causal variants mixed with a large number of noncausal variants; finally, gene-based tests coupled with functional annotation can facilitate the identification of extremely rare causal variants.

Recent developments in the multiple testing literature have introduced the false nondiscovery rate (Fndr). [115–117] We note that this is quite different from the AFNC control procedure. The Fndr controls for the expectation of the proportion FN/(*d* − *R*), which do not involve the number of causal variants *s* (see Table 1). Moreover, this is not a sensitive measure and will be very close to zero in large-scale NGS studies, as the number of variants that are not selected *d* − *R* will be very large. On the other hand, the AFNC, based on the proportion FN/*s*, allows robust variants selection in large-scale sequencing studies, as the number of causal variants *s* is expected to be small and the proportion FN/*s* is receptive to changes in the number of false negatives. In S7 Fig, we compared the AFNC with the Fndr at a threshold level of *β* = 0.1. Results suggest that the AFNC dominates the Fndr in terms of overall performances of *g*-measure and the Fndr performs poorly in terms of specificity.

Innovative technological advances have imposed new bioinformatic and statistical challenges by introducing genomic data at ever increasing resolution and dimensions. The proliferation of GWA studies in the last decade has largely led to the development and adaptation of the FDR as a conventional genomic tool. [42–46] In this paper, we introduced the AFNC to enable the identification of rare variants in large-scale sequencing studies. It is computationally efficient for applications in WGS and WES studies and can provide informative results for investigators charged with the task of analyzing large-scale sequencing studies.

## Methods

### Adaptive false-negative control of individual rare variants

The proposed procedure is general and can accommodate a spectrum of models and significance tests. Suppose that we have test statistics for each variant *T*_1_(*G*, *Z*), *T*_2_(*G*, *Z*), …, *T*_*d*_(*G*, *Z*) based on *i* = 1, 2, …, *n* subjects, such that *G* = {*G*_*ij*_} is a matrix of vectors of genotypes across all variants *j* = 1, 2, …, *d* and *Z* = {*Z*_*ik*_} is a matrix of vectors of additional covariates across various clinical factors and prior biological knowledge *k* = 1, …, *K*. Examples for *T*_*j*_(*G*, *Z*) include the classical *t*-test statistic that depends only on genotypes of the *j*th variant and the local FDR statistic that utilizes genotypes across all variants in an empirical Bayes construction. [108] Further, prior knowledge from functional annotation can be incorporated, such as by using a generalized linear mixed-effects model. [110] We assume that the test statistic *T*_*j*_(*G*, *Z*) for *j* = 1, 2, …, *d* is drawn from the mixture distribution

$$\begin{array}{c} \left(1-\pi \right)F_{0}+\pi F_{1}, \end{array}$$ (1)

where *π* = *s*/*d* is the signal proportion, *s* is the number of causal variants, *F*_0_ is the null distribution of *T*_*j*_(*G*, *Z*) when the *j*th variant is noncausal, and *F*_1_ is the alternative distribution when the *j*th variant is causal. [52, 53, 118] Let *T*_(1)_(*G*, *Z*) ≥ *T*_(2)_(*G*, *Z*) ≥ … ≥ *T*_(*d*)_(*G*, *Z*) be the ordered test statistics at decreasing significances.

To evaluate false negatives in NGS studies, we introduce the signal missing rate (SMR) for selecting the top *j* ranked variants as

$$\begin{array}{c} SMR^{\epsilon }\left(j\right)=P\left(FN(j)/s>\epsilon \right), \end{array}$$ (2)

where *FN*(*j*) is the number of causal variants missed by selecting the top *j* ranked variants and *ϵ* > 0 is a small constant. The SMR can be interpreted as the probability of neglecting at least a small proportion of causal variants among the top *j* ranked variants. By controlling the SMR, potentially causal variants can be included from both the Signals and Indistinguishable regions while dispatching with a very large number of irrelevant variants in the Noise region (see Fig 1). Compared to another possible measure of false negatives, *P*(*FN*(*j*)>0), SMR provides a more liberal control as it allows some, instead of zero, false negatives. SMR is also substantially different from the control of false nondiscovery rate (Fndr), which is an analog of FDR in terms of false negatives. Fndr is defined as the expectation of the proportion of false negatives among the accepted null hypotheses. [115, 119] In the analysis of data with extremely high dimensions and relatively small number of causal variants, Fndr is very close to zero and hence not an informative measure.

To provide informative analysis of rare variants in NGS studies, we propose the false-negative control screening (AFNC) procedure as follows.

1. Obtain ordered *p*-values from the test statistics *T*_(1)_(*G*, *Z*) ≥ *T*_(2)_(*G*, *Z*) ≥ … ≥ *T*_(*d*)_(*G*, *Z*) such that *p*_(1)_ ≤ *p*_(2)_ ≤ … ≤ *p*_(*d*)_.
2. Compute an estimate $\hat{\pi }$ of the signal proportion and compute $\hat{s}=\hat{\pi }d$.
3. Retain the top $\left\{1,2,\ldots ,{\hat{T}}_{fn}\right\}$ variants with
  $$\begin{array}{c} {\hat{T}}_{fn}=\left\{\begin{array}{cc} \hat{s} & \text{if}\;\hat{s}\leq t_{\alpha }\\ \hat{s}+\min\left\{j\geq 1:p_{\left(\hat{s}+j\right)}\leq F_{\hat{s},\left(j\right)}^{-1}\left(\beta \right)\right\} & \text{if}\;\hat{s}>t_{\alpha } \end{array}\right., \end{array}$$ (3)
  where $F_{\hat{s},\left(j\right)}^{-1}$ is the inverse cumulative distribution function of the *j*th ordered *p*-value among the $d-\hat{s}$ null (i.e., noncausal) variants; $F_{\hat{s},\left(j\right)}$ follows the Beta distribution with parameters *j* and $d-\hat{s}-j+1$; *t*_*α*_ is the cut-off position of the Bonferroni procedure at *α* significance level, and *β* is a pre-fixed level for controlling the signal missing rate. We set *α* and *β* at conventional levels of 0.05 and 0.1, respectively, in this paper. Smaller value of *β* corresponds to more stringent control on false negatives.

#### Step 2. Estimating π

To estimate the signal proportion *π* in Step 2, we employ the efficient estimator [50], based on empirical processes of *p*-values,

$$\begin{array}{c} \hat{\pi }=\max_{1<j<c_{0}d}\frac{j/d-p_{\left(j\right)}-c_{d}\sqrt{p_{\left(j\right)}\left(1-p_{\left(j\right)}\right)}}{1-p_{\left(j\right)}}, \end{array}$$ (4)

where 0 < *c*_0_ ≤ 1 is pre-fixed to accelerate the algorithm for large *d* by searching through only *c*_0_ fraction of the ranked variants. Conceptually, Eq (4) seeks for the largest difference between the observed, ordered *p*-value (i.e., *p*_(*j*)_) and the expected quantile under the global null (i.e., *j*/*d*). The largest difference typically occurs among the top proportion of the ranked *p*-values as causal variants tend to have small *p*-values. To ensure that we look through sufficient amount of top *c*_0_ *d* ordered variants (and hence the speed-up will have little impact on the results), we set a sufficiently large value for *c*_0_ *d*, i.e., at least 5000 or *d*/10, or equivalently, *c*_0_ *d* = max{5000, *d*/10}. The quantity *c*_*d*_ > 0 is pre-computed empirically to control the Type I error rate under the global null hypothesis that no causal variants exist. Specifically, we randomly simulate *M* sets of *p*-values, $p_{1,m}^{0},p_{2,m}^{0},\ldots ,p_{d,m}^{0}$, from the uniform distribution under the global null hypothesis for *m* = 1, 2, …, *M*. For set *m*, we order the *p*-values to obtain $p_{(1),m}^{0}\leq p_{(2),m}^{0}\leq \ldots \leq p_{(d),m}^{0}$, standardize them, and compute *V*_*m*_ by taking the maximum, i.e.,

$$\begin{array}{c} V_{m}=\max_{1<j\leq d}\left[\frac{j/d-p_{(j),m}^{0}}{\sqrt{p_{(j),m}^{0}\left(1-p_{(j),m}^{0}\right)}}\right]. \end{array}$$ (5)

Then, *c*_*d*_ is obtained as the (1 − *α*) quantile of the extreme values *V*_*m*_’s. Estimation of the signal proportion has been rigorously evaluated in the statistical literature. [50, 51, 120] In particular, under high dimensionality, statistical consistency of the estimator in Eq 4 does not depend on strict statistical normality assumptions and can be expected to perform well even when the proportion of causal variants *π* is very small. [50] It readily adapts to the underlying sparsity of the data in large-scale association studies.

#### Step 3. Obtaining the AFNC cut point ${\hat{T}}_{fn}$

The AFNC procedure evaluates statistical significance along the ordered *p*-values and retains the top ${\hat{T}}_{fn}$ variants of Eq 3 as important variants. When $\hat{s}\leq t_{\alpha }$, Eq 3 simplifies to ${\hat{T}}_{fn}=\hat{s}$ (which is ≤*t*_*α*_). In this case, if $\hat{s}>0$, the Bonferroni cut-off position *t*_*α*_ already encompasses the estimated number of causal variants. Such scenarios occur when the effect sizes are so strong that the Indistinguishable region degenerates in Fig 1 and nearly all causal variants can be identified in the Signals region. If $\hat{s}=0$, all variants are expected to be noncausal, which occurs under the global null hypothesis when both the Signals and Indistinguishable regions degenerate.

The more interesting scenario of $\hat{s}>t_{\alpha }$ occurs in NGS studies of rare variants when the Signals region is very small or degenerates and the Indistinguishable region may ensconce causal variants. In this case, we need to search further along the ordered test statistics, bypass some of the noncausal variants in the Indistinguishable region, and then stop when the number of false negatives is small relative to the total number of causal variants. The search starts at $\hat{s}$ and ends at the smallest *j*, $j=1,\ldots ,d-\hat{s}$, such that the observed *p*-values, $p_{\left(\hat{s}+j\right)}$, is no greater than the *β*-th quantile of the *j*-th ordered *p*-value, $P_{\left(j\right)}^{0}$, among the $d-\hat{s}$ null variants. The rationale is that when not all causal variants rank before $\hat{s}+j$, the number of noncausal variants among the top $\hat{s}+j$ variants, denoted by $n[\hat{s}+j]$, would be greater than *j*. Then the observed $p_{\left(\hat{s}+j\right)}$, which is in essence $\geq P_{n[\hat{s}+j]}^{0}$, would be greater than $P_{\left(j\right)}^{0}$. In other words, $p_{\left(\hat{s}+j\right)}>P_{\left(j\right)}^{0}$ is implied by the event that the top $\hat{s}+j$ variants still do not contain all causal variants. Therefore, our search should continue until the first time $p_{\left(\hat{s}+j\right)}\leq P_{\left(j\right)}^{0}$. In the extremely ideal case, one would wish that $Pr(P_{\left(j\right)}^{0}\geq p_{\left(\hat{s}+j\right)})\approx 1$. In real practice, we set $Pr(P_{\left(j\right)}^{0}\geq p_{\left(\hat{s}+j\right)})>1-\beta$ by looking for the *j* such that $p_{\left(\hat{s}+j\right)}$ is less than or equal to the *β*-th quantile of $P_{\left(j\right)}^{0}$ to achieve a better balance between a small false-negative proportion and a reasonable total number of variants selected. When this event occurs (i.e., $p_{\left(\hat{s}+j\right)}$ ≤ *β*-th quantile of $P_{\left(j\right)}^{0}$), the AFNC threshold ${\hat{T}}_{fn}$ asymptotically controls *SMR*^*ϵ*^ at level *β* for an arbitrarily small constant *ϵ* (i.e., *ϵ* is not changing with the total number of variants *d*).

In summary, using the cut-off position ${\hat{T}}_{fn}$, AFNC can adaptively encompass a large proportion (1 − *ϵ*) of the causal variants with high probability (≈1 − *β*). In the case where the causal and noncausal variants are better separated, ${\hat{T}}_{fn}$ of AFNC will become closer to the Bonferroni cut-off position *t*_*α*_. The AFNC procedure controls the signal missing rate with any consistent estimator of *π* (and in this paper, we employ the estimator of Eq 4). Finally, our procedure has a very low computational complexity *O*(*d* log *d*) and can be applied under extreme high dimensionality for WGS and WES studies.

## Supporting Information

S1 TextDerivation of signal missing rate control.We measure the false negatives using the signal missing rate (SMR) and show that SMR for ${\hat{T}}_{fn}$ can be asymptotically controlled at level *β*.(PDF)

S1 Fig Inclusion rate of causal variants across varying effect sizes and numbers of variants at *s* = 50. Success rates of including at least 50%, 75%, 90%, and 95% of *s* variants are examined. Results are shown for *s* = 50 number of causal variants when *C* ≠ 0 and *n* = 2000 number of samples.
(PDF)

S2 Fig Comparisons across varying effect sizes and numbers of variants at *s* = 25. Performance of AFNC, FDR, and Bonferroni is evaluated in terms of sensitivity, specificity, and *g*-measure. Results are shown for *s* = 25 number of causal variants when *C* ≠ 0 and *n* = 2000 number of samples.
(PDF)

S3 Fig Inclusion rate of causal variants across varying effect sizes and numbers of variants at *s* = 25. Success rates of including at least 50%, 75%, 90%, and 95% of *s* variants are examined. Results are shown for *s* = 25 number of causal variants when *C* ≠ 0 and *n* = 2000 number of samples.
(PDF)

S4 Fig Inclusion rate of causal variants across sample sizes and numbers of causal variants at *C* = 0.5. Success rates of including at least 50%, 75%, 90%, and 95% of *s* variants are examined. Results are shown for the effect-size multiplier *C* = 0.5 and *d* = 100,000 number of variants.
(PDF)

S5 Fig Comparisons across varying sample sizes and numbers of causal variants at *C* = 0.25. Performance of AFNC, FDR, and Bonferroni is evaluated in terms of sensitivity, specificity, and *g*-measure. Results are shown for the effect-size multiplier *C* = 0.25 and *d* = 100,000 number of variants.
(PDF)

S6 Fig Inclusion rate of causal variants across sample sizes and numbers of causal variants at *C* = 0.25. Success rates of including at least 50%, 75%, 90%, and 95% of *s* variants are examined. Results are shown for the effect-size multiplier *C* = 0.25 and *d* = 100,000 number of variants.
(PDF)

S7 Fig Comparisons across varying effect sizes and numbers of variants at *s* = 50 with the Fndr. Performance of AFNC, FDR, and Bonferroni is compared with that of the Fndr in terms of sensitivity, specificity, and *g*-measure. Results are shown for *s* = 50 number of causal variants when *C* ≠ 0 and *n* = 2000 number of samples.
(PDF)

S1 Table Empirical type I error rates across varying sample sizes. Standard errors are included in parentheses. Results are shown for *d* = 100,000 number of variants.
(PDF)

S2 Table Full annotation of AFNC-selected variants in the analysis of CoLaus data. Gene-set *p*-values are computed using the SKAT. Genes are sorted in alphabetic order, and variants are sorted by their individual *p*-values among each gene. Variants marked with (*) are also selected by the FDR.
(PDF)

S1 File Files for simulations and analysis of CoLaus data. File “simulation_code.R” contains R code for simulations. SNPs used to generate phenotypes at *n* = 2000 are included as “snp.n2000.RData”. File “CoLaus_code.R” contains R code for the analysis of CoLaus data.
(ZIP)

S1 Dataset Single-locus and gene-level *p*-values used in the analysis of CoLaus data. Dataset “single_locus_pvalues.txt” contains variant-level *p*-values used in the analysis of the CoLaus data. Dataset “gene_level_pvalues.txt” contains gene-level *p*-values computed from the SKAT.
(ZIP)
