# Supplementary material for: Rare Variants Association Analysis in Large-Scale Sequencing Studies at the Single Locus Level
Source: PLoS Comput Biol. 2016 Jun 29;12(6):e1004993. doi: 10.1371/journal.pcbi.1004993 (PMC4927097; doi:10.1371/journal.pcbi.1004993)
Supplement: S1 Text — We measure the false negatives using the signal missing rate (SMR) and show that SMR for T^fn can be asymptotically controlled at level β. (PDF) [file pcbi.1004993.s001.pdf]

S1 Text

In this section, we will establish that

$$SMR(\hat{T}_{fn}) \leq \beta + o(1). \tag{S1}$$

Define $\hat{j} = \min\{j \geq 1 : \; p_{(\hat{\pi}d+j)} \leq F^{-1}_{\hat{\pi},(j)}(\beta)\} 1_{\{\hat{\pi}d > t_\alpha\}}$, then $\hat{T}_{fn} = \hat{\pi}d + \hat{j}$. Also define $n(j) = j - s(j)$ as the number of noncausal variants among the top j ranked variants. We have

$$\begin{aligned}
SMR(\hat{T}_{fn}) &= P(s(\hat{T}_{fn}) < (1-\epsilon)s) \\
&= P(n(\hat{T}_{fn}) > \hat{T}_{fn} - (1-\epsilon)s) \\
&= P(n(\hat{T}_{fn}) > \hat{\pi}d + \hat{j} - (1-\epsilon)\pi d) \\
&\leq P(n(\hat{T}_{fn}) > \hat{\pi}d + \hat{j} - (1-\epsilon)\pi d, \hat{\pi} \geq (1-\epsilon)\pi) + P(\hat{\pi} < (1-\epsilon)\pi) \\
&\leq P(n(\hat{T}_{fn}) > \hat{j}) + o(1),
\end{aligned} \tag{S2}$$

where the second equality is by $\hat{T}_{fn} = n(\hat{T}_{fn}) + s(\hat{T}_{fn})$, the third equality is by $s = \pi d$, and the last step is by the consistency of $\hat{\pi}$.

When $\hat{\pi}d \leq t_\alpha$, we have $\hat{j} = 0$ and $\hat{T}_{fn} = \hat{\pi}d$. Then

$$P(n(\hat{T}_{fn}) > \hat{j}) = P(n(\hat{\pi}d) > 0) \leq P(n(t_\alpha) > 0) = \alpha \tag{S3}$$

When $\hat{\pi}d > t_\alpha$, denote $P^0_{(j)}$ as the jth ordered p-value of $n - s$ noncausal variants, then

$$\begin{aligned}
P(n(\hat{T}_{fn}) > \hat{j}) &= P(P^0_{(\hat{j})} < p_{(\hat{T}_{fn})}) \\
&\leq P(P^0_{(\hat{j})} < F^{-1}_{\hat{\pi},(\hat{j})}(\beta)) \\
&\leq P(P^0_{(\hat{j})} < F^{-1}_{\hat{\pi},(\hat{j})}(\beta), \hat{\pi} \leq \pi) + P(\hat{\pi} > \pi) \\
&\leq P(P^0_{(\hat{j})} < F^{-1}_{\pi,(\hat{j})}(\beta)) + o(1).
\end{aligned}$$

By the definition of F^{-1}, (S1) follows.
