# Supplementary material for: Rare Variants Association Analysis in Large-Scale Sequencing Studies at the Single Locus Level
Source: PLoS Comput Biol. 2016 Jun 29;12(6):e1004993. doi: 10.1371/journal.pcbi.1004993 (PMC4927097; doi:10.1371/journal.pcbi.1004993)
Supplement: S3 Fig — Success rates of including at least 50%, 75%, 90%, and 95% of s variants are examined. Results are shown for s = 25 number of causal variants when C ≠ 0 and n = 2000 number of samples. (PDF) [file pcbi.1004993.s004.pdf]

S3 Fig

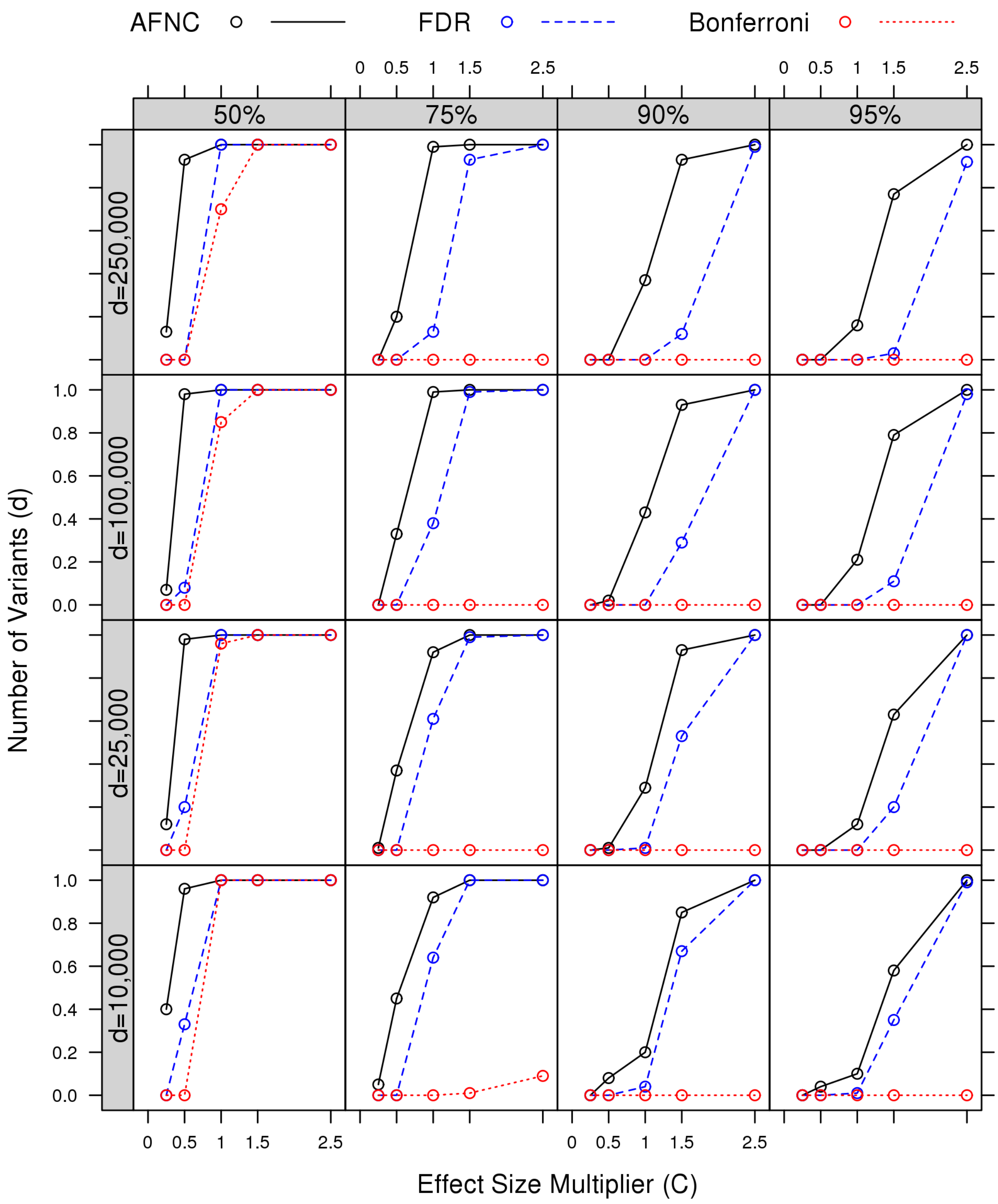

Figure S3: **Inclusion rate of causal variants across varying effect sizes and numbers of variants at $s = 25$.** Success rates of including at least 50%, 75%, 90%, and 95% of s variants are examined. Results are shown for $s = 25$ number of causal variants when $C \neq 0$ and $n = 2000$ number of samples.
