# Supplementary material for: Rare Variants Association Analysis in Large-Scale Sequencing Studies at the Single Locus Level
Source: PLoS Comput Biol. 2016 Jun 29;12(6):e1004993. doi: 10.1371/journal.pcbi.1004993 (PMC4927097; doi:10.1371/journal.pcbi.1004993)
Supplement: S4 Fig — Success rates of including at least 50%, 75%, 90%, and 95% of s variants are examined. Results are shown for the effect-size multiplier C = 0.5 and d = 100,000 number of variants. (PDF) [file pcbi.1004993.s005.pdf]

S4 Fig

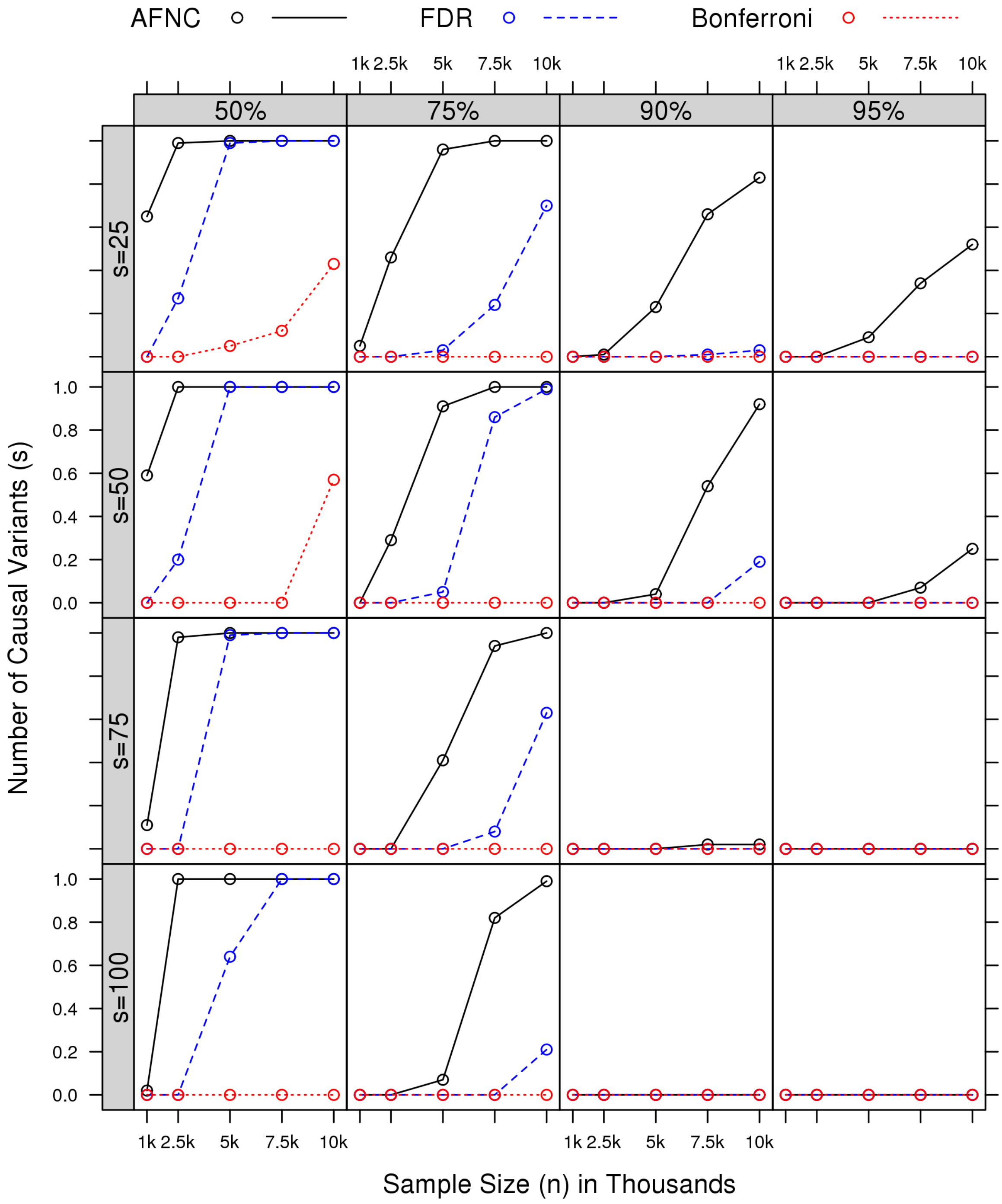

Figure S4: **Inclusion rate of causal variants across sample sizes and numbers of causal variants at $C = 0.5$.** Success rates of including at least 50%, 75%, 90%, and 95% of s variants are examined. Results are shown for the effect size multiplier $C = 0.5$ and $d = 100,000$ number of variants.
