# Supplementary material for: Rare Variants Association Analysis in Large-Scale Sequencing Studies at the Single Locus Level
Source: PLoS Comput Biol. 2016 Jun 29;12(6):e1004993. doi: 10.1371/journal.pcbi.1004993 (PMC4927097; doi:10.1371/journal.pcbi.1004993)
Supplement: S5 Fig — Performance of AFNC, FDR, and Bonferroni is evaluated in terms of sensitivity, specificity, and g-measure. Results are shown for the effect-size multiplier C = 0.25 and d = 100,000 number of variants. (PDF) [file pcbi.1004993.s006.pdf]

S5 Fig

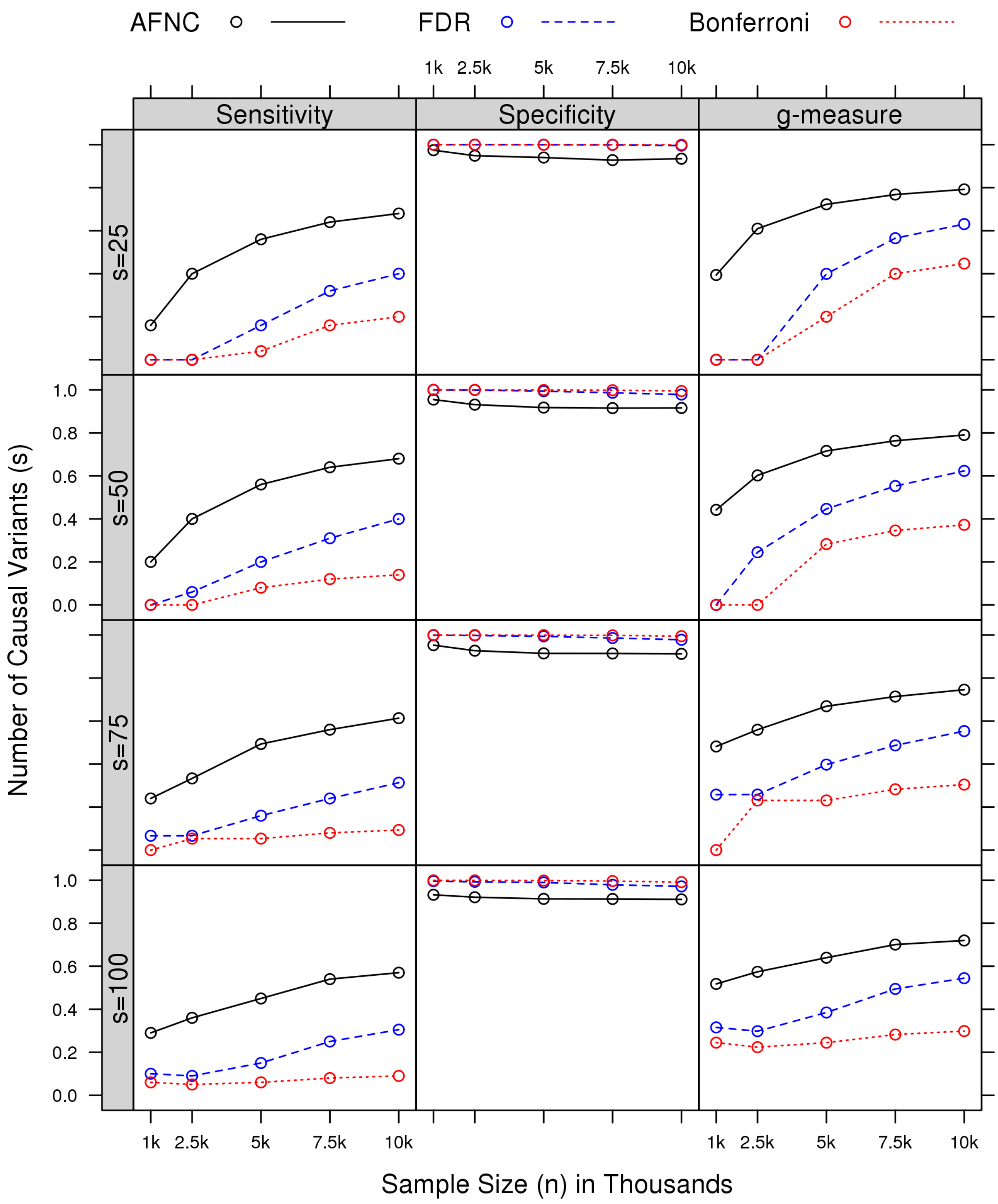

Figure S5: **Comparisons across varying sample sizes and numbers of causal variants at $C = 0.25$.** Performance of AFNC, FDR, and Bonferroni is evaluated in terms of sensitivity, specificity, and g-measure. Results are shown for the effect size multiplier $C = 0.25$ and $d = 100,000$ number of variants.
