# Supplementary material for: Rare Variants Association Analysis in Large-Scale Sequencing Studies at the Single Locus Level
Source: PLoS Comput Biol. 2016 Jun 29;12(6):e1004993. doi: 10.1371/journal.pcbi.1004993 (PMC4927097; doi:10.1371/journal.pcbi.1004993)
Supplement: S1 Table — Standard errors are included in parentheses. Results are shown for d = 100,000 number of variants. (PDF) [file pcbi.1004993.s009.pdf]

S1 Table

Sample size	Bonferroni	FDR	AFNC
n=1000	0 (2.43×10^{-6})	0 (1.01×10^{-4})	0.006 (0.030)
n=2500	0 (8.05×10^{-6})	0 (4.58×10^{-5})	0.008 (0.031)
n=5000	0 (0)	0 (2.85×10^{-5})	0.002 (0.031)
n=7500	0 (3.15×10^{-6})	0 (5.71×10^{-5})	0.003 (0.026)
n=10,000	0 (0)	0 (2.23×10^{-5})	0.001 (0.028)

Table S1: **Empirical type I error rates across varying sample sizes**. Standard errors are included in parentheses. Results are shown for $d = 100,000$ number of variants.
