# Supplementary material for: Rare Variants Association Analysis in Large-Scale Sequencing Studies at the Single Locus Level
Source: PLoS Comput Biol. 2016 Jun 29;12(6):e1004993. doi: 10.1371/journal.pcbi.1004993 (PMC4927097; doi:10.1371/journal.pcbi.1004993)
Supplement: S2 Table — Gene-set p-values are computed using the SKAT. Genes are sorted in alphabetic order, and variants are sorted by their individual p-values among each gene. Variants marked with (*) are also selected by the FDR. (PDF) [file pcbi.1004993.s010.pdf]

S2 Table

Gene (gene-set p-value)	Variant ID	Variant p-value	Variant type
ADORA1 (0.159)	chr1_201364975	5.06×10^{-4}	upstream
APH1A (1.90×10^{-3})	*chr1_148504677	5.15×10^{-6}	downstream
APP (0.23)	chr21_26291512	2.06×10^{-4}	intron
BRD2 (0.281)	chr6_33053682	2.08×10^{-3}	non-synonymous coding
CCR9 (0.218)	chr3_45917527	6.44×10^{-4}	synonymous coding
CDH2 (0.229)	chr18_23784893	2.55×10^{-3}	upstream
CLEC16A (0.0902)	chr16_11125133	2.06×10^{-4}	non-synonymous coding
	chr16_11125102	2.06×10^{-4}	synonymous coding
	chr16_10970541	1.39×10^{-3}	synonymous coding
CNR2 (0.139)	chr1_24073736	2.27×10^{-3}	non-synonymous coding
CYSLTR2 (0.0748)	chr13_48180709	1.09×10^{-3}	3' UTR
	chr13_48180617	1.39×10^{-3}	3' UTR
EGR1 (0.136)	chr5_137832662	3.16×10^{-3}	upstream
HCRTR1 (0.763)	chr1_31861752	2.07×10^{-3}	synonymous coding
HHIP (0.747)	chr4_145847791	5.06×10^{-4}	synonymous coding
IL18 (0.469)	chr11_111539886	1.81×10^{-3}	upstream
ITGA4 (0.34)	*chr2_182029869	5.15×10^{-6}	upstream
ITGB1 (0.729)	chr10_33255067	3.14×10^{-3}	downstream
KCNN4 (0.456)	chr19_48965473	6.44×10^{-4}	non-synonymous coding
KIAA1967 (0.989)	chr8_22529454	3.16×10^{-3}	downstream
LRRK2 (0.532)	chr12_39048760	9.23×10^{-5}	downstream
MAG (0.236)	chr19_40477034	6.21×10^{-5}	intron
MME (0.387)	chr3_156315473	1.03×10^{-3}	splice site
NLRP1 (0.303)	chr17_5425965	2.73×10^{-3}	non-synonymous coding
NTRK2 (0.231)	chr9_86619100	3.16×10^{-3}	3' UTR
OPRM1 (0.627)	chr6_154454129	5.06×10^{-4}	non-synonymous coding
P4HB (0.938)	chr17_77394570	2.55×10^{-3}	downstream
PDE4A (0.313)	chr19_10439268	2.06×10^{-4}	non-synonymous coding
PDE5A (0.114)	chr4_120635588	1.81×10^{-3}	downstream
PSEN1 (0.221)	chr14_72672989	2.69×10^{-3}	upstream
PTGS1 (0.494)	chr9_124195307	2.27×10^{-3}	downstream
PYGB (0.395)	chr20_25205884	1.81×10^{-3}	synonymous coding
RORC (0.411)	chr1_150054386	2.78×10^{-3}	synonymous coding
RTN4 (0.0803)	chr2_55063212	6.21×10^{-5}	synonymous coding
	chr2_55131218	2.27×10^{-3}	intron
SCN9A (0.291)	chr2_166845210	6.21×10^{-5}	non-synonymous coding
SDHB (0.674)	chr1_17232220	9.24×10^{-4}	non-synonymous coding
SIRT5 (0.31)	chr6_13720352	2.27×10^{-3}	upstream
SIRT6 (6.68×10^{-3})	chr19_4125175	2.06×10^{-4}	3' UTR
SLC5A1 (0.0191)	*chr22_30792914	5.15×10^{-6}	intron
	chr22_30769209	9.40×10^{-4}	5' UTR
SP110 (4.14×10^{-3})	chr2_230785852	6.12×10^{-4}	non-synonymous coding
	chr2_230745800	1.17×10^{-3}	splice site
STIM1 (0.519)	chr11_4069349	6.01×10^{-4}	synonymous coding
SYK (0.0803)	chr9_92676970	2.07×10^{-3}	intron
	chr9_92667248	3.16×10^{-3}	intron
TACR3 (0.0149)	chr4_104859945	2.06×10^{-4}	non-synonymous coding
TLR4 (0.299)	chr9_119518750	6.21×10^{-5}	downstream
	chr9_119519234	2.07×10^{-3}	downstream
TNNI3K (0.537)	chr1_74701758	1.25×10^{-3}	non-synonymous coding
TRPC6 (0.55)	chr11_100828104	2.55×10^{-3}	3' UTR
TRPM8 (3.54×10^{-3})	*chr2_234559154	5.15×10^{-6}	non-synonymous coding
	chr2_234543736	6.21×10^{-5}	non-synonymous coding
	chr2_234556441	6.44×10^{-4}	synonymous coding
	chr2_234591833	6.63×10^{-4}	downstream

ZAP70 (0.0636)	chr2_97696780	9.24×10^{-4}	intergenic

Table S2: **Full annotation of AFNC-selected variants in the analysis of CoLaus data.** Gene-set p-values are computed using the SKAT. Genes are sorted in alphabetic order, and variants are sorted by their individual p-values among each gene. Variants marked with (*) are also selected by the Bonferroni and FDR.
